# School Anthropometry

**Published:** 1906-03-31

**Authors:** 


### The Hospital.

A Journal of

The Medical Sciences and Hospital Administration.

Vol. XXXIX.—No. 1,018. SATURDAY, MARCH 31, 1906.

### SCHOOL ANTHROPOMETRY.

The Bishop of Ripon, writing to the *Times* as Chairman of the National League for Physical Education and Improvement, makes an earnest appeal to public opinion in support of the demand of the League for a complete, systematic, and compulsory medical inspection of schools, an inspection which should include the ascertainment and record, at least once a year, of the height, weight, and chest-measurement of every child; observation of the condition of eyes, ears, nose, and throat; the detection of any disease, deformity, or defect of general health; and the employment of all possible means to remove the causes and to prevent the diffusion of offensive or infectious disease among children. It appears from the Bishop's letter that a deputation from the League was received last month by Mr. Birrell, the President of the Board of Education, and that among those composing it there were distinguished members of the medical profession—Sir Lauder Brunton, Professor Howard Marsh, and Mrs. Scharlieb; while the Episcopal Bench, the Jewish community, and the Salvation army were also represented. It is, perhaps, not surprising that Mr. Birrell, while expressing his personal sympathy with the objects of the deputation, nevertheless pointed out the difficulties arising from expense and from the possible prejudices of parents; and somewhat clearly indicated that increased pressure from the public would be an indispensable preliminary of any legislative action in the direction indicated. He seems to have made it clear that the Government would not commit themselves to a costly project unless they were fully assured that it was one which would be likely to receive general support and approval. The Bishop therefore accepts the gauntlet which the Minister has thrown down, and endeavours to establish a case in favour of the administrative action which he desires. He pleads, on the authority of Professor Marsh, that medical inspection would do much to banish such ailments as measles, diphtheria, and scarlet fever, "as completely as rabies has been banished by recent measures." He pleads that the future of many children would be happier and more useful to society if, as a result of medical inspection, they were persuaded not to enter occupations for which they are physically unfit; and, further, that the inspection would result in economy to the nation, on the ground that neglected infantile ailments lead to waste of human power, and in the end cast upon society the burden of supporting those who might have been rendered able to support themselves. Finally he urges that "prevention" is at once better, more economical, and more humane than "cure," and he cites for the instruction of his fellow-countrymen the example of what has been done elsewhere. He tells us that there are already 300 school doctors in New York, 130 in Paris, and as many as 8,000 in Japan; and he adds that the benefits to society and to the children are beyond doubt.

We have the greatest possible respect for th philanthropy and the good intentions of the Bishop, who somewhat reminds us of the pious wish of Henry IV., that every Frenchman should have a fowl in his pot. We should welcome the desired inspection, notwithstanding its inevitable cost, if we saw any indication that the evils which it might disclose would be dealt with by measures primarily directed against negligent or criminal parents. The whole tendency of modern action has been to diminish the sense of parental responsibility; and it is high time to reconsider the position, and to make parents understand that, if society owes any duty to them, they also have a reciprocal duty to society, and one which society is prepared to enforce, if necessary, by strong measures. We have so far had scarcely any mean between absolute neglect and misdirected philanthropy; and it would, perhaps, be hard to say which of the two is calculated to be the more injurious to the parents concerned and to the community. The increased attention which is now being directed towards questions of public health, and towards the physical value of children as a national asset, must necessarily lead us to reconsider a good many accepted notions, the acceptance of which, in many cases, has been due more to intellectual indolence than to careful consideration. We have lately seen a good deal in the newspapers about the formation of a "middle-class party" as a new political organisation; and it is quite clear that its efforts would be directed, in the first place, towards resistance against many demands for so-called improvements which were to be effected mainly at the cost of one section of the community and mainly for the real or supposed advantage of another. Questions of education and of public health have been so far dealt with as if it were the business of the clerk and the small tradesman to provide these benefits for the so-called "working classes," and the aforesaid working classes have succeeded in convincing many politicians that they are the only persons to be considered in modern legislation. Indications are not wanting that the sorely overloaded camel is already looking askance at the straws which episcopal and other reformers are preparing as additions to his burden.

